# SCMR president's page

**DOI:** 10.1186/1532-429X-12-23

**Published:** 2010-04-09

**Authors:** Eike Nagel

**Affiliations:** 1 Division of Imaging Sciences, King's College London, BHF Centre of Research Excellence, NIHR Biomedical Research Centre; Wellcome Trust and EPSRC Medical Engineering Centre, The Rayne Institute, St. Thomas Hospital, London, SE1 7EH, UK

## Abstract

News from the Society for Cardiovascular Magnetic Resonance

## Editorial

The year 2010 has started well for SCMR in many aspects. The 2010 Annual Scientific Sessions were a great success with 1138 participants, the highest ever for our Annual Meeting. Andrew Arai, MD as Programme Chair and Sven Plein, MD as Abstract Chair did a terrific job in putting together a sound scientific program with excellent posters, scientific presentations and invited talks. As a novelty, an additional track was introduced, featuring clinical cases for discussion, which was highly welcomed by the participants. We will try to strengthen these case-based sessions in our future meetings. We are looking forward to the next meeting February 3-6, 2011 in Nice, France and hope to be able to attract many known and unknown faces. SCMR has awarded five regional scholarships for the 2010 meeting. We will continue to do so for next year, further expand the scheme and also have an additional scholarship specifically for technologists starting in 2011. Please stay tuned to our web pages for details.

I would like to highlight our award winners 2009/2010:

**Young Investigator Award Clinical**: Tim Lockie - Division of Imaging Sciences, King's College London, UK for *Detection of hemodynamically significant coronary stenoses with k-t SENSE-accelerated Myocardial Perfusion MR Imaging at 3.0 Tesla - a comparison with fractional flow reserve*

**Young Investigator Award Experimental**: Andrew Flett - The Heart Hospital, London, UK for *Equilibrium contrast MR for the measurement of diffuse myocardial fibrosis *and Cameron Holloway - University of Oxford, UK for *Normobaric hypoxia elevates free fatty acids and impairs cardiac energetics and diastolic function in normal human volunteers*.

**Moderated Poster Clinical**: Andreas Kumar - University of Calgary, Canada for *Reperfusion hemorrhage is a marker for the severity of tissue injury in patients with acute ST-elevation myocardial infarction*

**Moderated Poster Experimental**: Ronald Beyers - University of Virginia, USA for *Whole-heart T2-weighted (T2w) sequence for imaging post-infarct edematous area at risk (AR) in mice*

**Best Technologist Abstract**: Saundra B. Grant - Allegheny General Hospital, USA for *The concordance rates between left ventricular hypertrophy and right ventricular hypertrophy in patients with hypertrophic cardiomyopathy as diagnosed by CMR with fibrosis imaging*

**Best Web Case**: Ethan Ellis (Harvard), Boston, USA for *All That Elevates Is Not Plaque Rupture*

**Gerald Pohost JCMR Best Manuscript Award**: Stamatios Lerakis - Emory University, USA for *Prognostic value of adenosine perfusion CMR*

A special thank you to Dwaine Rieves, MD from the FDA, who attended all three days of the Scientific Sessions and presented during the Board meeting and the Clinical Trials/Clinical Practice Committee meetings. We heard with interest that there are new possibilities to get gadolinium chelates approved for cardiac imaging and that our efforts to standardize data acquisition [1] and reporting [2] were regarded as major steps towards strengthening the role of CMR in clinical trials. We will further improve our standards by preparing a document for standardized data post-processing.

Thanks to Chris Kramer, MD our immediate Past President as well as Scott Flamm, MD and Mark Fogel, MD, SCMR is well represented in the multiple activities led by the American College of Cardiology (ACC) and American College of Radiology (ACR) for developing guidelines and Appropriateness Criteria for different imaging modalities.

There was some sad news on the rejection of a project proposal put forward by Drs. Kramer, Schulz-Menger and Nagel to compare CMR with echocardiography for the prediction of cardiac events and remodeling by the NIH. The feedback we are getting from various funding bodies is to design trials, which change outcome, rather than proving superior diagnosis alone.

Our regional chapters have shown enormous activities over the last year. Most notably, the Latin American chapter has developed a lively webpage as an excellent resource, and the European Chapter (mainly through the Euro-CMR working group of the European Society of Cardiology) has started data acquisition for a multi-national CMR registry (Euro-CMR registry) which we hope to expand world-wide in the upcoming months.

The education committee chaired by Victor Ferrari, MD, has developed a range of training cases acquired in accordance to SCMR standards and published them on our webpages. James Moon, MD, and his team have managed to videotape almost all presentations from the meeting and published them on the web at the time of the meeting. For those who have not visited our webpages for a while it is definitely worth to do this soon, as there is a wealth of new information assembled.

New committee chairs assumed their positions following the annual meeting. They include: Clinical Practice - Steve Wolff; Clinical Trials - Matthias Friedrich; Education - Timothy Christian; Financial - Albert de Roos; Membership - Harold Litt; Public Relations - Victor Ferrari; Publications - Nat Reichek; Science - Krishna Nayak; and Technologist - Susan Eder. The WWW Committee will be led again by Michael Jerosch-Herold. The 2011 Annual Scientific Sessions Program Chair is Sven Plein, and the 2011 Co-chair and Abstract Chair is Raymond Kwong.

JCMR, the Society's Journal of CMR, is growing stronger since transitioning from a hard print journal to the open access online format. The impact factor for JCMR rose to 2.15 in 2009 from 1.87 in 2008. There were 56 papers published in 2009, approximately one per week. SCMR will pay the author publishing charge for current members, an outstanding membership benefit.

SCMR is enjoying a phase of stability in its management with a strong team from Talley in place (Deborah Berkowitz, Executive Director, Kathy Baumer, Meeting Manager, as well as others), which is continuously, and actively supporting all our activities, the most obvious being the yearly meeting, which ran smoothly, despite considerable difficulties due to the temporary closure of Phoenix International Airport.

Plans for the upcoming year contain strengthening our links to ISMRM as there is a unique chance of streamlining activities between the two societies with Debiao Li, PhD being a member of the Board of Trustees of SCMR and the incoming president of ISMRM. We also hope to strengthen our ties to the (yet underrepresented) French CMR community and will work on ways to allow colleagues from less developed countries to join SCMR.

SCMR seems to have weathered the financial storms of last year without major damage. This is due to our own efforts to reduce our costs, as well as the continuous support of our Platinum supporters Siemens and Philips as well as support from General Electric, and, Toshiba.

I would like to use this chance to thank our Immediate Past President Chris Kramer, MD for passing over the Society in such great shape. Dr. Kramer has advertised CMR in many areas to its best advantage, has finally made the Nice meeting possible, and has strengthened our voice significantly. I am looking forward to serving SCMR for the next year, embracing the different disciplines and nationalities involved in this ever-moving field and hope to be able to maintain the momentum and further strengthen CMR and the Society. Please contact me for any comment or idea you might have, or activity you would like to perform within SCMR.
